# Apatinib Suppresses Gastric Cancer Stem Cells Properties by Inhibiting the Sonic Hedgehog Pathway

**DOI:** 10.3389/fcell.2021.679806

**Published:** 2021-07-19

**Authors:** Wanshuang Cao, Yuan Li, Hongliang Sun, Chenying Yang, Jianyun Zhu, Chunfeng Xie, Xiaoting Li, Jieshu Wu, Shanshan Geng, Lu Wang, Liangfei Sun, Guozhu Geng, Hongyu Han, Caiyun Zhong

**Affiliations:** ^1^Cancer Research Division, Center for Global Health, School of Public Health, Nanjing Medical University, Nanjing, China; ^2^Department of Clinical Nutrition, Nanjing Drum Tower Hospital, The Affiliated Hospital of Nanjing University Medical School, Nanjing, China; ^3^Department of Urology, Taikang Xianlin Drum Tower Hospital, The Affiliated Hospital of Nanjing University Medical School, Nanjing, China; ^4^Suzhou Digestive Diseases and Nutrition Research Center, Suzhou Municipal Hospital, The Affiliated Suzhou Hospital of Nanjing Medical University, Suzhou, China; ^5^Jiangsu Hengrui Medicine Co., Ltd., Lianyungang, China; ^6^State Key Laboratory of Oncology in South China, Department of Clinical Nutrition, Collaborative Innovation Center for Cancer Medicine, Sun Yat-sen University Cancer Center, Guangzhou, China

**Keywords:** apatinib, gastric cancer stem cells, sonic hedgehog pathway, suppression, chemoresistance

## Abstract

The presence of gastric cancer stem cells (GCSCs) marks the onset of gastric carcinoma. The sonic hedgehog (SHH) pathway plays a vital role in the maintenance of GCSC characteristics. Apatinib has been approved in China for advanced gastric cancer (GC) treatment. However, whether apatinib can target GCSCs and affect the SHH pathway remains unclear. The present study aimed to investigate the underlying mechanism of apatinib’s antitumor effects on GC. The expression levels of GCSC markers and number of CD133^+^ cells were significantly elevated in the sphere-forming cells. Apatinib effectively suppressed GCSC traits by inhibiting tumorsphere formation and cell proliferation, suppressing GCSC markers expression and CD133^+^ cell number, and inducing apoptosis. Apatinib downregulated the activation of the SHH pathway; while upregulation of the SHH pathway attenuated the inhibitory effects of apatinib on GCSCs. Moreover, apatinib treatment significantly delayed tumor growth and inhibited GCSC characteristics in the xenograft model. Our data suggested that apatinib exhibited inhibitory effects on GCSCs by suppressing SHH pathway both *in vitro* and *in vivo*, thus providing new insights into the therapeutic application of apatinib in GCSC suppression and advanced gastric cancer treatment.

## Introduction

Gastric cancer (GC) is the fifth most common (5.7%) cancer and the third leading cause (8.2%) of cancer-related mortality worldwide, and more than 60% of all cases occur in eastern and southeastern Asia (Bray et al., 2018). At diagnosis, almost two-thirds of patients present with locally advanced or metastatic disease because the early GC stages are clinically silent (Lordick et al., 2014). Since early 1990s, neoadjuvant therapies, including chemotherapy, have been more frequently applied for locally advanced or initially irresectable GC (D’Ugo et al., 2009). Although palliative chemotherapy can induce tumor regression in some patients, recurrence is common and recurrent cancer is highly chemoresistant (Li et al., 2015). Consequently, the median overall survival (OS) is still poor (Lordick et al., 2017). Hence, more effective therapies against GC relapse are urgently needed.

Apatinib (AiTan, China), also known as rivoceranib, is an oral small-molecule tyrosine kinase inhibitor (Tian et al., 2011) which highly selectively binds to vascular endothelial growth factor receptor 2 (VEGFR-2) and suppresses tumor angiogenesis (Jayson et al., 2016). A phase III trial (Li et al., 2016) revealed that compared with placebo, apatinib significantly prolonged median progression-free survival and OS in patients with advanced GC or gastroesophageal junction adenocarcinoma for whom at least two lines of prior chemotherapy had failed. Epidemiological evidence indicates that apatinib monotherapy is preferred as a third-line therapy for advanced esophagogastric cancer (Ter Veer et al., 2016). Apatinib has also exhibited promising therapeutic efficacy against epithelial ovarian cancer (Miao et al., 2018), glioma (Wang et al., 2017), colorectal cancer (Chen et al., 2019) and hepatocellular carcinoma (Lu et al., 2017) in clinical trials; however, the molecular mechanisms underlying its antitumor activity remain to be elucidated.

Cancer stem cells (CSCs) are a subpopulation of cells within a tumor with capacities for self-renewal, differentiation, metastatic dissemination, drug resistance, and tumor recurrence (Vermeulen et al., 2008). Gastric cancer stem cells (GCSCs), first described in 2007 by Yang et al. (2007), are the root of GC development and progression (Takaishi et al., 2009; Singh, 2013; Li et al., 2014; Brungs et al., 2016; Molina-Castro et al., 2017). Chemoresistance is an intrinsic characteristic of CSCs and arises from various mechanisms, including upregulation of drug-efflux pumps, DNA damage repair, and other prosurvival and antiapoptotic effects (Borst, 2012; Zhao, 2016). Chemotherapy can not only increase the fraction of side-population (SP) cells (Tsuchida et al., 2008), but also promote the self-renewal of CSCs (Debeb et al., 2012). Thus, targeting GCSCs can be an effective GC therapeutic strategy, especially after post-chemotherapy relapse and recurrence.

The maintenance of CSCs, including GCSCs, occurs through the aberrant activation of the sonic hedgehog (SHH) pathway, thus contributing to cancer development and progression (Ruiz i Altaba et al., 2007; Song et al., 2011; Kim et al., 2017). The SHH pathway is initiated by the binding of SHH protein to its receptor Patched (PTCH). When the SHH pathway is inactive, PTCH suppresses signal transduction by inhibiting the transmembrane protein Smoothened (Smo). Upon SHH binding, the inhibitory effect is relieved, resulting in the nuclear translocation of glioma-associated oncogenes (Gli) transcription factors, eventually regulating the downstream genes related to cell stemness, proliferation and apoptosis. In addition, the combination of classical chemotherapeutic drugs with altering Gli code exhibits an additive or synergistic effect (Clement et al., 2007; Feldmann et al., 2007; Yin et al., 2018).

Although the inhibitory effect of apatinib on angiogenesis has been well established, whether and how it affects GCSCs remains unclear. Therefore, we investigated the suppressive action of apatinib on GCSCs and the role of SHH pathway in this process.

## Materials and Methods

### Cell Culture and Reagents

Gastric cancer cell lines BGC-823 and SGC-7901 were purchased from the Chinese Academy of Typical Culture Collection Cell Bank (Shanghai, China). Both cell lines were maintained in RPMI-1640 medium (Gibco, Grand Island, NY, United States) supplemented with 10% fetal bovine serum (FBS) (Gibco) and 1% antibiotics (penicillin/streptomycin) (Gibco) at 37°C with 5% CO_2_.

Apatinib was obtained from Jiangsu Hengrui Medicine (Lianyungang, China). Purmorphamine was acquired from Selleckchem (Houston, TX, United States). Vismodegib was purchased from MCE (Junction, NJ, United States). The growth factors, epidermal growth factor (EGF), basic fibroblast growth factor (bFGF), and insulin were obtained from Peprotech (Rocky Hill, NJ, United States), and 2% B27 was obtained from Gibco.

### Tumorsphere Formation Assay

Tumorspheres were generated by seeding BGC-823 and SGC-7901 cells in six-well ultralow attachment plates (1 × 10^5^ cells per well) with a serum-free DMEM/F12 medium (SFM) (Gibco). The SFM of BGC-823 cells was supplemented with 5 μg/L EGF and 2% B27, and the medium of SGC-7901 included 20 μg/L bFGF, 20 μg/L EGF, 2% B27, and 5 μg/mL insulin. The spheroids were grown for 6 days, and the medium were refreshed every 48 h. The images of the representative fields were captured using a bright-field microscope (Nikon, Tokyo, Japan).

To analyze the effect of apatinib on GCSCs, both sphere-forming cells were treated with different concentrations of apatinib (0, 1, 2, 5, and 10 μM). Six days after treatment, the tumorspheres of each group were imaged.

### Western Blotting

Tumorspheres were collected and lysed in RIPA buffer (Beyotime, Shanghai, China) containing protease inhibitors. Protein concentration was measured using the BCA protein assay kit (Pierce, Rockford, WI, United States). Total protein (40–60 μg per sample) was then loaded on sodium dodecyl sulfate gel electrophoresis and transferred onto nitrocellulose filter membranes (PALL, NY, United States). After blocking, the membranes were incubated with primary antibodies overnight at 4°C, followed by horseradish peroxidase-conjugated secondary antibodies (ZSGB-BIO, Beijing, China). Glyceraldehyde 3-phosphate dehydrogenase (GAPDH) was served as the internal control. Antibodies for CD133, CD44, Oct4, Sox2, Nanog, epithelial cellular adhesion molecule (EpCAM), P-glycoprotein (P-gp), ABCC1, VEGFR-2, SHH, Smo, Gli1, Gli2, Proliferating Cell Nuclear Antigen (PCNA), Cyclin D1, Bcl-2, Bax, Cleaved Caspase (8,9,3), and GAPDH were purchased from Proteintech (Rosemont, IL, United States). Antibody for p-VEGFR-2 (Tyr1175) was purchased from Affinity Biosciences Inc. (Cincinnati, OH, United States). All antibody information is presented in Supplementary Table 1.

### Immunofluorescence Staining

The cell spheroids were washed with phosphate-buffered saline (PBS) supplemented with 0.5% Tween20 (PBST) and fixed in methyl alcohol. Subsequently, the spheroids were blocked with 5% bovine serum albumin for 2 h at room temperature. Cells were then incubated with rabbit anti-CD133 and EpCAM antibodies (dilution 1:100) at 4°C overnight. After being washed with PBST, cells were incubated with cy3-conjugated goat antirabbit IgG (dilution 1:200) (Beyotime). 4′,6-Diamidino-2-phenylindole (DAPI) (Sigma, St. Louis, MO, United States) was used to stain the nucleus for 15 min. The fluorescent images were captured using a fluorescence microscope (Olympus IX-70, Tokyo, Japan).

### EdU Assay

Cells were seeded into 96 well culture plates (1 × 10^4^ cells per well) and EdU assay was performed with BeyoClick^*TM*^ EdU Cell Proliferation Kit with Alexa Fluor 488 according to the manufacturer’s instructions (Beyotime). Cell fluorescence was detected use a High Content Screening instrument (ThermoFisher ArrayScan VTI, United States).

### Transient Transfection

BGC-823 and SGC-7901 cells were seeded into six-well plates at a density of 2 × 10^5^ cells in RPMI-1640 medium containing 10% FBS without antibiotics. Transfection of EX-NEG-M29-Gli1 (2 μg) and the corresponding control vector EX-NEG-M29 (2 μg) was performed by Lipofectamine 3000 reagent (Invitrogen, Carlsbad, CA, United States). After 6 h, cells were trypsinized and then cultured in SFM overnight. Subsequently, cells were treated with or without apatinib for another 4 days. Cell lysates were used to measure the indicated protein levels.

### Colony Formation Assay

The sphere-forming cells were suspended and separated into single cells. Cells were seeded into six-well plates (500 cells per well) and cultured in RPMI-1640 medium containing 10% FBS, antibiotics and different concentrations of apatinib for the other 13 days. Colonies were fixed with 10% cold formaldehyde for 10 min, followed by incubating with crystal violet at room temperature for 10 min. Stained cells were then photographed under a microscope (Olympus, Tokyo, Japan).

### Cell Viability

The cell spheroids were treated with the indicated concentrations of apatinib. After 6 days of incubation, cell viability was evaluated using a cell counting kit-8 (CCK-8) assay (Beyotime) according to the manufacturer’s instructions. The absorbance at 450 nm was quantified on a multimode reader (Infinite M200 Pro; Tecan, Männedorf, Switzerland).

### Detection of CD133^+^ Cells Through Flow Cytometry

After collection by centrifugation, the adherent cells and cell spheroids were washed twice with PBS. A 1 × 10^6^ single-cell suspension was stained with 1 μL of APC-conjugated human monoclonal anti-CD133 antibody (Miltenyi Biotech, Teterow, Germany) at 4°C for 10 min in the dark. Immunoglobulin G (IgG) isotype antibody (Miltenyi Biotec) was used as a negative control. The stained samples were analyzed using the FACS Aria III system (BD Biosciences, San Jose, CA, United States).

### Detection of Apoptotic Cells Through Flow Cytometry

After exposure to different concentrations of apatinib for 4 days, the sphere-forming cells were harvested and resuspended in 400 μL of binding buffer. The cells were then stained with 5 μL of Annexin V-FITC at 4°C in the dark for 15 min, followed by incubation with 5 μL of propidium iodide (PI) at room temperature in the dark for 5 min. Subsequently, the stained cells were subjected to flow cytometry within 1 h. Both PI-negative/Annexin V-positive and PI-positive/Annexin V-positive cells were defined as apoptotic cells.

### Hoechst 33258 Staining

Sphere-forming cells were treated with apatinib at various concentrations. After treatment, the fixed cells were stained with 5 μg/mL Hoechst 33258 solution (Beyotime) according to the manufacturer’s instructions. The tumorspheres were then visualized under a reverse fluorescence microscope (Olympus IX-70) with excitation at 350 nm and emission at 460 nm.

### Apatinib Treatment in Xenograft Model

Fifteen 4-week-old BALB/c nude mice (male) were purchased from the Animal Core Facility of Nanjing Medical University (Nanjing, China). The animals received humane care, and all experiments were performed in accordance with the guidelines of the Animal Care and Welfare Committee of Nanjing Medical University (IACUC-1907002). 5 × 10^6^ BGC-823 cells were suspended in 0.1 mL of PBS and implanted subcutaneously into the right flank region of nude mice. Body weight and tumor volume were measured every other day. The tumor volume (V) was calculated as 0.5 × length × (width)^2^. To determine the effects of apatinib on tumor growth, 15 mice bearing gastric tumors of approximately 140 mm^3^ (7 days after implantation) were randomized into three groups: (a) vehicle-only solution, (b) 50 mg/kg apatinib, and (c) 100 mg/kg apatinib. Both vehicle and apatinib suspended in carboxymethylcellulose sodium salt were administered using oral gavage once daily for 14 days. Subsequently, the mice were killed, and tumor tissues were collected and fixed in 4% formalin or stored at −80°C for further analysis.

### Immunohistochemistry

A series of 4-μm-thick sections were obtained from each paraffin block. The sections were incubated with rabbit antibodies against CD44, Sox2, EpCAM, VEGFR-2, Ki67, PCNA, and P-gp (dilution 1:200) as well as a mouse antibody against Gli1 (dilution 1:100) overnight at 4°C; all these primary antibodies were purchased from Proteintech. Detailed antibody information is presented in Supplementary Table 1. The sections were then incubated with goat anti-rabbit/mouse biotin at room temperature for 1 h. Signals were amplified with a Vectastain Elite ABC Kit (Vector Laboratories, Burlingame, CA, United States). Finally, the slides were scanned using a pannoramic MIDI scanner. For each animal, three tumor sections were analyzed.

### Online Database

An online database of gene expression profiling interactive analysis (GEPIA, Peking University, Beijing, China^1^) (Tang et al., 2017) was used to evaluate target gene expression in tumors and normal gastric tissues, and in different tumor stages. Gene expression correlation analysis was performed for given sets of The Cancer Genome Atlas (TCGA) expression data.

The online Kaplan–Meier plotter database (KM plotter^2^) was applied to assess the prognostic values [OS, first progression (FP), and post progression survival (PPS)] of target genes in 875 clinical GC cases (Szász et al., 2016). Patients with GC were divided into high and low expression groups according to the median expression of target genes. The association between particular genes and survival outcomes was analyzed through the data of RNA-seq and gene chip through Kaplan–Meier survival curves. Hazard ratios with their 95% confidence intervals and log-rank *P* value were evaluated on website.

### Statistical Analysis

Data are presented as mean ± standard deviation (SD). All experiments were performed in triplicates. Statistical differences between two groups were determined using a two-tailed Student *t* test. One-way analysis of variance was conducted among multiple groups. Densitometric values were quantified by Image J software. GraphPad Prism (version 6.0) was used for statistical analysis. Statistical significance was set at a *p* value of <0.05.

## Results

### Evidence for Clinical Significance and Prognostic Value of GCSCs and SHH Related Genes

We firstly analyzed the expression of classic GCSCs marker genes and key molecules of the SHH pathway using TCGA online database. We found that the mRNA expression levels of several GCSCs markers and Gli were increased in GC tissues (*n* = 408) compared with normal tissues (*n* = 211; Supplementary Figure 1). Upregulation of Gli1 in tumor group was significantly associated with the late stage of GC (Supplementary Figure 1). We further investigated whether these target genes were correlated to the prognosis of patients with GC. The prognostic value of these genes was obtained from K-M plotter. It was shown that higher mRNA expression levels of several GCSCs related genes, especially the key components of SHH signal pathway (SHH, Smo, Gli1 and Gli2), were significantly associated with worse OS, FP and PPS (Supplementary Figure 2). Although these data were based on mRNA expression profiling, our results suggested that SHH signaling may be a key player in advanced GC that needs to be further explored.

### Enrichment of GCSCs by SFM Culture *in vitro*

**FIGURE 1:**
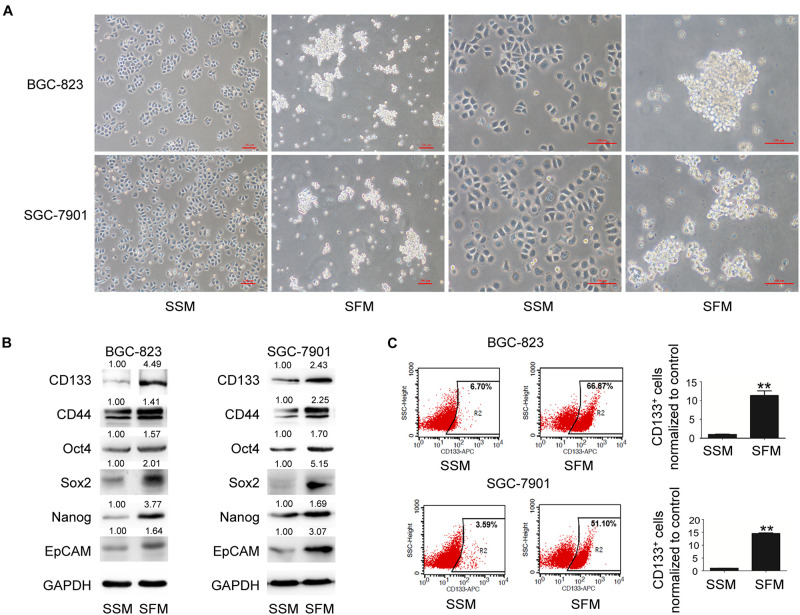
GCSC tumorsphere formation in SFM culture. BGC-823 and SGC-7901 cells were separately cultured in SSM and SFM for 6 days. **(A)** Cell morphology was imaged under a light microscope. Bar 100 μm. **(B)** Protein expression of GCSC markers, including CD133, CD44, Oct4, Sox2, Nanog, and EpCAM, were determined using Western blotting. **(C)** Detection of CD133^+^ cells in both cells through flow cytometry. Data are presented as the mean ± SD of three independent experiments. **p < 0.01 compared with SSM group.

The tumorsphere formation assay is a well-accepted method for the isolation and enrichment of CSC populations. In the present study, both BGC-823 and SGC-7901 cells formed tumorspheres (diameter > 50 μm) with SFM culture (Figure 1A). As illustrated in Figure 1B, 6 days after SFM culture, the protein levels of GCSC markers, including CD133, CD44, Oct4, Sox2, Nanog, and EpCAM, were upregulated compared with the adherent counterparts culturing in serum supplied medium (SSM). In addition, flow cytometry analysis indicated a significantly increased percentage of CD133^+^ cells in these sphere-forming cells (a 10-fold increase in BGC-823 and a 14-fold increase in SGC-7901 compared with SSM; Figure 1C and Supplementary Figure 3). Collectively, these results suggested the enrichment of GCSCs from BGC-823 and SGC-7901 cells through tumorsphere formation assay.

### Apatinib Suppressed the Stemness of GCSCs

**FIGURE 2:**
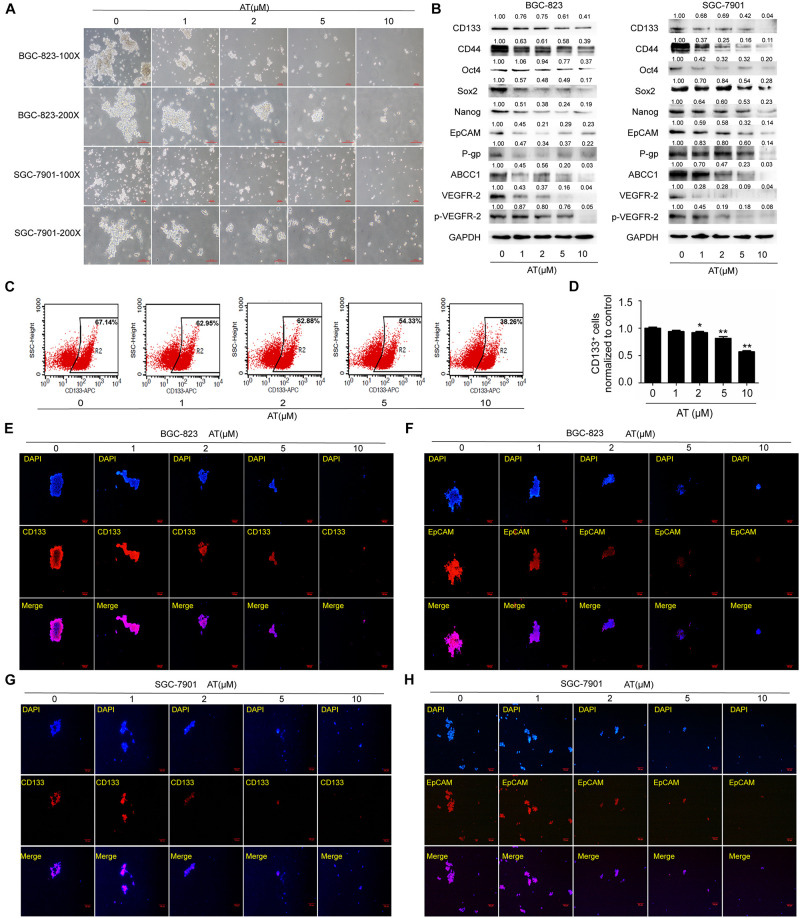
Inhibition of GCSC traits by apatinib. Tumorsphere-forming cells were treated with different concentrations of apatinib for 6 days. **(A)** Representative images of sphere-forming cells. Bar 100 μm. **(B)** Protein levels of GCSC markers (CD133, CD44, Oct4, Sox2, Nanog, and EpCAM), P-gp, ABCC1, VEGFR-2 and p-VEGFR-2 were determined using Western blotting. **(C,D)** Detection of CD133^+^ cells in BGC-823 spheroids through flow cytometry. **(C)** Representative images. **(D)** Percentage of CD133^+^ cells. **(E,F)** Immunofluorescence staining images of BGC-823 spheroids were obtained to determine CD133 and EpCAM expression. **(G,H)** Immunofluorescence staining images were obtained to determine CD133 and EpCAM expression in SGC-7901 spheroids. Data are presented as the mean ± SD of three independent experiments. *p < 0.05, **p < 0.01 compared with the control group.

We then examined the effects of apatinib on enriched GCSC spheroids. The spheroids were treated with various apatinib concentrations (0, 1, 2, 5, and 10 μM). Apatinib dose-dependently reduced the spheroid size (Figure 2A). As apatinib concentration increased, the protein expression of GCSCs markers gradually reduced in the spheroids of both cell lines. Apatinib treatment downregulated drug resistance proteins (P-gp, ABCC1), VEGFR-2 and p-VEGFR-2 (Figure 2B). Moreover, the percentage of CD133^+^ cells was decreased after apatinib treatment (Figures 2C,D). Immunofluorescence staining also demonstrated similar results for CD133 and EpCAM protein expression in both cell lines (Figures 2E–H and Supplementary Figures 4A,B). Taken together, these data revealed that apatinib inhibited the traits of GCSCs.

### Apatinib Inhibited GCSC Proliferation

**FIGURE 3:**
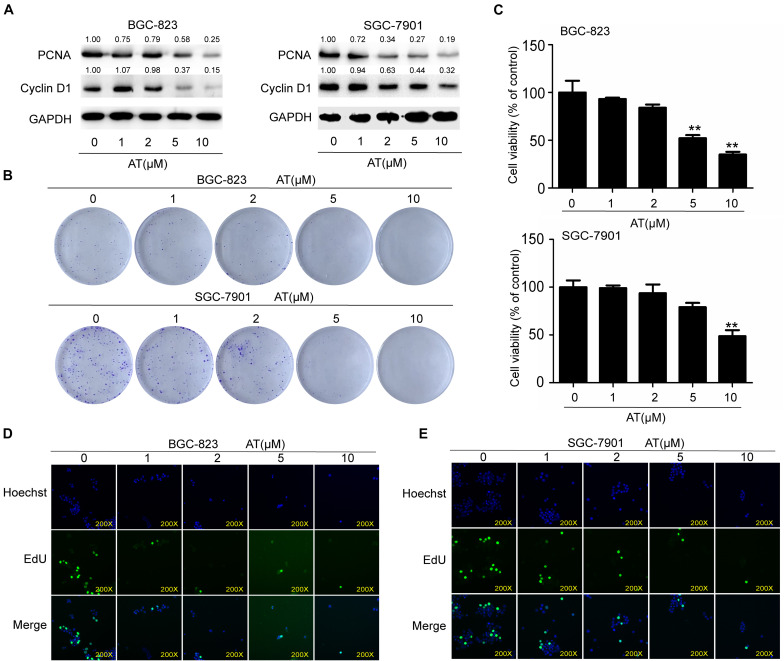
Suppression of GCSC proliferation by apatinib. BGC-823 and SGC-7901 spheroids were incubated with appropriate concentrations of apatinib for 6 days. **(A)** Protein levels of PCNA and Cyclin D1 were measured through Western blotting. **(B)** Colony formation ability was assessed with a colony formation assay. **(C)** Cell viability was measured using CCK-8. **(D,E)** EdU assay was used to determine the ability of apatinib on gastric cancer cells growth (magnification, 200×). Data are presented as the mean ± SD of three independent experiments. **p < 0.01 compared with the control group.

To further investigate the inhibitory effects of apatinib, we examined GCSC proliferation following apatinib treatment. Apatinib considerably reduced the expression of cell cycle and cell proliferation related proteins PCNA and Cyclin D1 (Figure 3A). Apatinib inhibited colony formation in both BGC-823 and SGC-7901 cells (Figure 3B). CCK-8 assay showed that 10 μM apatinib significantly reduced the cell viability of BGC-823 and SGC-7901 sphere-forming cells to 35.04 ± 2.92% and 48.64 ± 6.38% of control, respectively (Figure 3C). In addition, EdU assay showed that the proliferation of BGC-823 and SGC-7901 sphere-forming cells was decreased by apatinib (Figures 3D,E). These results suggested that apatinib suppressed GCSC proliferation.

### Apatinib Induced GCSC Apoptosis

**FIGURE 4:**
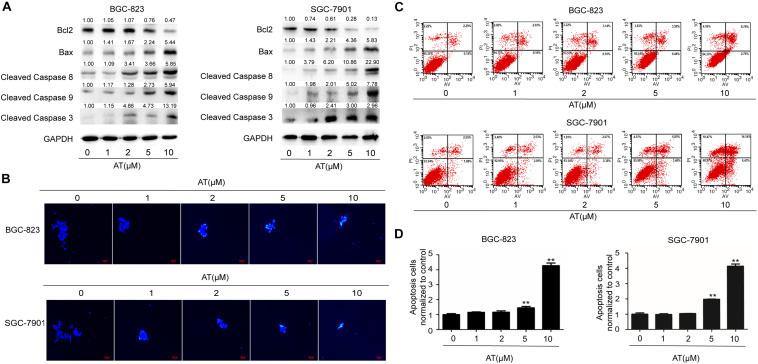
Induction of GCSC apoptosis by apatinib. Cell spheroids were treated with apatinib (1–10 μM) for 4 days and fed every 24 h. **(A)** The protein levels of apoptosis-related proteins were measured using Western blotting. **(B)** Hoechst 33258 staining of the tumorspheres. Bar 100 μm. **(C,D)** The percentage of apoptotic cells was analyzed with flow cytometry. **(C)** Representative images. **(D)** Percentage of apoptotic cells. Data are presented as the mean ± SD of three independent experiments. **p < 0.01 compared with the control group.

Next we examined the effect of apatinib on GCSC apoptosis. Decreased level of anti-apoptotic protein Bcl2 as well as elevated levels of pro-apoptotic proteins Bax and Cleaved Caspases were observed (Figure 4A). Hoechst 33258 staining revealed apoptosis in sphere-forming cells following apatinib treatment (Figure 4B and Supplementary Figure 5A). Furthermore, flow cytometry analysis indicated that apatinib increased the apoptosis rate in both BGC-823 and SGC-7901 cells (Figures 4C,D and Supplementary Figure 5B). Together, these data illustrated that apatinib induced GCSC apoptosis.

### Apatinib Suppressed SHH Pathway in GCSCs

**FIGURE 5:**
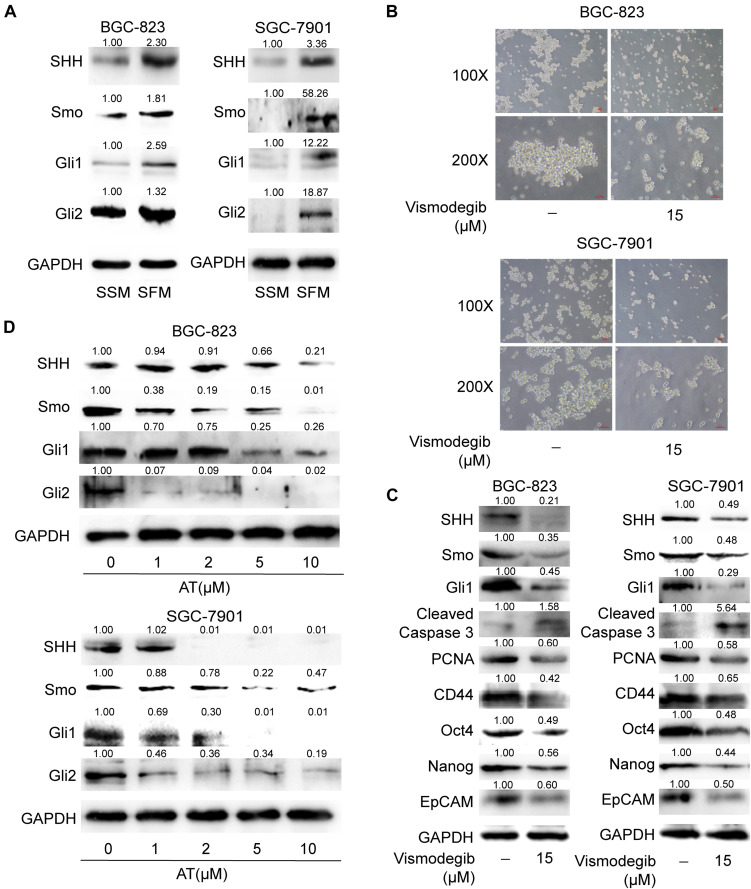
Suppression of the SHH pathway in GCSCs by apatinib. **(A)** Cells cultured in SSM and SFM were collected, and changes in SHH, Smo, Gli1, and Gli2 levels were measured through Western blotting. The spheroids were incubated with vismodegib (15 μM) for 6 days. **(B)** Representative images (magnification, 100× and 200×). **(C)** The protein levels of critical molecules (SHH, Smo and Gli1) in the SHH pathway, Cleaved Caspase 3, PCNA and GCSC markers were detected through Western blotting. **(D)** The tumorspheres were treated with 1–10 μM apatinib for 6 days, and the expression of SHH pathway components was analyzed by Western blotting.

Given that SHH pathway is critical in maintaining CSC stemness (Ruiz i Altaba et al., 2007), we evaluated whether apatinib influenced the activation of SHH pathway in GCSCs. Our results revealed that the SHH pathway components, including SHH, Smo, Gli1, and Gli2, were considerably upregulated in the sphere-forming cells (Figure 5A). We also found that downregulation of SHH activation by vismodegib, a Smo inhibitor, resulted in suppression of tumorsphere formation ability, decrease of GCSC markers expression, reduced PCNA expression and increased cleaved caspase 3 level (Figures 5B,C). Furthermore, apatinib significantly decreased the protein expression of SHH pathway molecules in the sphere-forming cells (Figure 5D). Thus, these results suggested that apatinib inhibited the activation of the SHH pathway in GCSCs.

### Apatinib Inhibited GCSC Properties Through SHH Pathway Suppression

**FIGURE 6:**
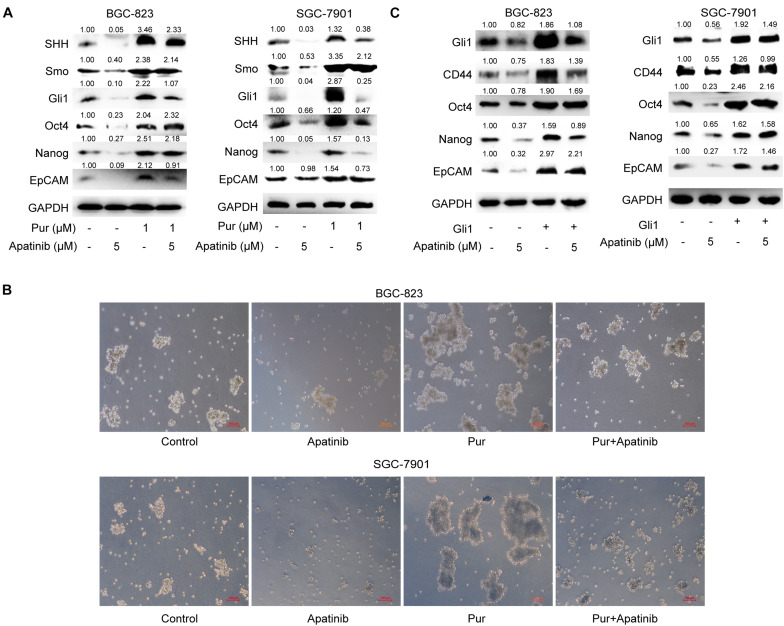
Downregulation of the SHH pathway mediates the inhibitory effects of apatinib on GCSCs. **(A,B)** Sphere-forming cells were incubated with 5 μM apatinib with/without 1 μM purmorphamine for 6 days. **(A)** The protein levels of SHH pathway molecules were measured using Western blotting. **(B)** Representative images. Bar 100 μm. **(C)** Both cells were transfected with a control vector or Gil1 plasmid (2 μg). Following 24 h of transfection, cells were then treated with or without apatinib (5 μM) for another 4 days. The levels of GCSC markers were detected by Western blotting. Pur, purmorphamine.

We next explored the role of SHH pathway in the inhibitory effects of apatinib on GCSCs. Purmorphamine, a Smo activator, was used to activate SHH pathway. We showed that purmorphamine facilitated tumorsphere formation and upregulated the levels of GCSCs markers. The suppressive effects of apatinib on tumorsphere formation, SHH pathway and GCSC markers were attenuated when purmorphamine was coadministered with apatinib (Figures 6A,B). Moreover, transfection of Gli1 plasmids enhanced GCSC activity and abolished apatinib induced downregulation of GCSC markers in sphere-forming cells (Figure 6C). Taken together, these results indicated that SHH pathway plays an essential role in the inhibitory effects of apatinib on GCSCs.

### Apatinib Inhibited GCSC Traits in Xenograft Model

**FIGURE 7:**
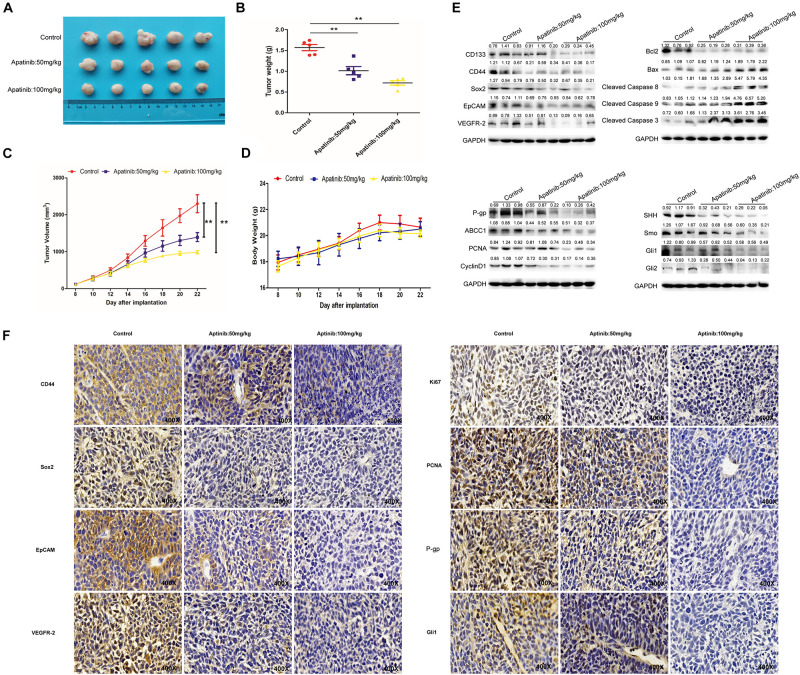
Inhibition of GCSCs traits by apatinib *in vivo*. The xenograft model exerted antitumor efficacy *in vivo*. **(A)** Tumor size 22 days after implantation. **(B)** Changes in tumor weight. Data are presented as the mean ± SD. **(C)** Changes in tumor volume. Data are presented as mean ± SD. **(D)** Changes in body weight. Data are presented as the mean ± SD. **(E)** Protein levels of GCSC markers, drug-resistant proteins, VEGFR-2, proliferation-related proteins, apoptosis-related proteins, and SHH pathway molecules were determined through Western blotting. **(F)** Expression of GCSC markers, drug-resistant proteins, VEGFR-2, proliferation-related proteins, apoptosis-related proteins, and Gli1 was measured using immunohistochemistry (magnification, 400×). **p < 0.01 compared with the control group.

Finally, a xenograft model was established to determine the effects of apatinib on gastric tumor *in vivo*. Apatinib suppressed gastric tumor growth in a dose-dependent manner (Figures 7A–C and Supplementary Figure 6A). After 14 days of apatinib treatment, the terminal tumor weight was significantly reduced (Figure 7B). Compared with the control group, the tumor volume of the 100 mg/kg apatinib group was remarkably decreased by 1.3-fold (from 2,299.51 ± 541.10 mm^3^ to 979.34 ± 90.99 mm^3^) (Figure 7C and Supplementary Figure 6A). Meanwhile, no significant difference was observed in mice body weight during apatinib treatment (Figure 7D). The protein expression levels of GCSC markers, VEGFR-2, and drug resistance proteins, were reduced with apatinib treatment (Figure 7E and Supplementary Figures 6B,D). In addition, apatinib treatment increased apoptosis-related proteins expression, decreased cell proliferation-related proteins expression, and inhibited SHH pathway proteins expression in gastric tumors (Figure 7E and Supplementary Figures 6C–E). Similar results were revealed by immunohistochemistry assays (Figure 7F). Thus, these data illustrated that apatinib repressed GCSC traits and the SHH pathway *in vivo*, which was in line with our *in vitro* results.

## Discussion

Despite advances in chemotherapy and surgery, the prognosis of patients with advanced GC remains poor. Chemotherapy could induce the enrichment of GCSCs, which are highly associated with the degree of malignancy and drug resistance (Shaheen et al., 2016). Apatinib has been approved as third-line treatment for metastatic GC in China. Regarding the anticancer mechanisms of apatinib, most research has focused on antiangiogenesis. In the present study, we revealed that apatinib suppressed GCSCs properties and downregulated SHH pathway both *in vitro* and *in vivo*.

Serum-free medium culture, which is based on the capability of CSCs to form three-dimensional spheres under serum-free culture conditions *in vitro*, has been widely used for CSCs isolation and enrichment (Shaheen et al., 2016). Combined detection of CD133 and CD44 expression has been used for the clinical prediction and diagnosis of GC (Chen et al., 2013; Brungs et al., 2016). EpCAM(+)/CD44(+) cells grow exponentially *in vitro* as cancer spheres with SFM culture (Han et al., 2011). Furthermore, pluripotent stem cell factors, such as Oct4, Sox2, and Nanog, also participate in the regulation of GCSCs properties (Yong et al., 2016; Molina-Castro et al., 2017). In the present study, both BGC-823 and SGC-7901 cells cultured in SFM had elevated tumorsphere formation capacity, considerably increased expression levels of GCSCs markers and increased percentage of CD133^+^ cells in sphere-forming cells, indicating the enrichment of GCSCs.

Non-stem cancer cells can acquire stemness under some conditions, including chemotherapy (Hu et al., 2012; Li et al., 2015). CSCs are subpopulations of cells within tumors that can maintain tumor growth, metastasis, chemoresistance, and recurrence (Singh, 2013). As the first generation of oral antiangiogenesis drug approved in China, apatinib has demonstrated acceptable safety, tolerability, and efficacy in the treatment of advanced GC (Li et al., 2016; Zhang et al., 2017), which agrees with the results of our xenograft model. Our data revealed no difference in mice body weight between the control group and apatinib treatment group. Apatinib can target SP cells to enhance the efficacy of conventional chemotherapeutical drugs in leukemia cells (Tong et al., 2012). However, its anti-GCSCs properties remained unclear. Therefore, in the present study we determined the effects of apatinib on GCSCs characteristics. Our findings showed that apatinib effectively inhibited GCSCs properties by suppressing tumorsphere formation capacity, GCSCs marker expression, drug-resistant protein expression, VEGFR-2 and p-VEGFR-2 expression, and the number of CD133^+^ cells. Moreover, apatinib suppressed GCSCs proliferation and induced GCSCs apoptosis.

SHH pathway plays a pivotal role in stemness maintenance and tumorigenesis (Cochrane et al., 2015). After SHH pathway is activated, transcription factors Gli transmit the cytoplasmic signal and enhance the transcription of downstream target genes (Kinzler et al., 1988). Dysregulation of SHH pathway is frequently identified in GC (Wan et al., 2014; Akyala and Peppelenbosch, 2018). Moreover, SHH critically participates in maintaining GCSCs characteristics (Song et al., 2011; Samadani and Akhavan-Niaki, 2015; Kim et al., 2017). Consistent with these findings, we demonstrated that SHH pathway was highly activated in GCSCs, whereas SHH suppression abolished GCSCs traits, suggesting the essential role of SHH pathway in GCSC stemness.

Thus far, the role of SHH pathway in the suppressive effects of apatinib on GCSCs remains unclear. We demonstrated that apatinib inhibited SHH pathway in GCSCs and that the activation of SHH by purmorphamine and Gli1 plasmid transfection attenuated the effects of apatinib on tumorsphere formation, GCSCs marker expression, and SHH pathway activation in GCSCs. Collectively, these results indicated that apatinib suppressed GCSCs by inhibiting SHH pathway. Similarly, our *in vivo* study also revealed that apatinib treatment repressed GCSCs traits and SHH pathway. Nevertheless, further research is warranted to investigate the detailed mechanisms underlying apatinib’s regulation of the SHH pathway.

## Conclusion

In summary, our study demonstrated that apatinib inhibited the properties of GCSCs by downregulating SHH pathway. These novel findings could provide critical information for the therapeutic application of apatinib in GCSC suppression and GC treatment.

## Data Availability Statement

The original contributions presented in the study are included in the article/Supplementary Material, further inquiries can be directed to the corresponding author/s.

## Ethics Statement

The animal study was reviewed and approved by the Animal Care and Welfare Committee of Nanjing Medical University.

## Author Contributions

CZ, HH, and GG conceived and designed the study. WC, YL, HS, CY, JZ, CX, XL, JW, SG, LW, and LS conducted the experiments and analyzed the data. WC, YL, and CZ wrote the manuscript. HH and CZ provided the funding of this study. CZ had primary responsibility for the final content. All authors have read and approved the final manuscript.

## Conflict of Interest

LW, LS, and GG are employees of Jiangsu Hengrui Medicine Co., Ltd. The remaining authors declare that the research was conducted in the absence of any commercial or financial relationships that could be construed as a potential conflict of interest.

## Footnotes

1 http://gepia.cancer-pku.cn/

2 http://kmplot.com/analysis/

## Abbreviations

CSCs: cancer stem cells
GCSCs: gastric cancer stem cells
SHH: sonic hedgehog
PTCH: patched
Smo: smoothened
Gli: glioma-associated oncogene
VEGFR-2: vascular endothelial growth factor receptor 2
FBS: fetal bovine serum
EGF: epidermal growth factor
bFGF: basic fibroblast growth factor
SFM: serum-free medium
GAPDH: glyceraldehyde 3-phosphate dehydrogenase
EpCAM: epithelial cellular adhesion molecule
P-gp: p glycoprotein
PBST: phosphate buffer saline with Tween20
BSA: bovine serum albumin
DAPI: 4′, 6-diamidino-2-phenylindole
CCK8: cell counting kit-8
PI: propidium iodide
V: volume
SD: standard deviation
SSM: serum supplied medium.
