# Supplementary material for: Apatinib Suppresses Gastric Cancer Stem Cells Properties by Inhibiting the Sonic Hedgehog Pathway
Source: Front Cell Dev Biol. 2021 Jul 19;9:679806. doi: 10.3389/fcell.2021.679806 (PMC8326764; doi:10.3389/fcell.2021.679806)
Supplement: Supplementary file 1 [file Data_Sheet_1.docx]

***Supplementary materials***

**Tables of contents**

Supplementary Table 1.

Supplementary Figure 1.

Supplementary Figure 2.

Supplementary Figure 3.

Supplementary Figure 4.

Supplementary Figure 5.

Supplementary Figure 6.

**Supplementary Table 1**

| **Antibody** | **Catalog number** | **Application in the present study** |
| --- | --- | --- |
| CD133 | 18470-1-AP | WB, IF |
| CD44 | 15675-1-AP | WB, IHC |
| Oct4 | 11263-1-AP | WB |
| Sox2 | 11064-1-AP | WB, IHC |
| Nanog | 14295-1-AP | WB |
| EpCAM | 21050-1-AP | WB, IHC, IF |
| P-gp | 22336-1-AP | WB, IHC |
| ABCC1 | 67228-1-Ig | WB |
| VEGFR-2 | 26415-1-AP | WB, IHC |
| p- VEGFR-2 | AF4426 | WB |
| SHH | 20697-1-AP | WB |
| Smo | 20787-1-AP | WB |
| Gli1 | 66905-1-Ig | WB, IHC |
| Gli2 | 18989-1-AP | WB |
| PCNA | 10205-2-AP | WB, IHC |
| Cyclin D1 | 26939-1-AP | WB |
| Bcl-2 | 12789-1-AP | WB |
| Bax | 50599-2-Ig | WB |
| Cleaved Caspase 3 | 19677-1-AP | WB |
| Cleaved Caspase 8 | 13423-1-AP | WB |
| Cleaved Caspase 9 | 10380-1-AP | WB |
| GAPDH | 10494-1-AP | WB |
| Ki67 | 27309-1-AP | IHC |

**Supplementary Table 1.** Information of antibodies used in the present study.

**Supplementary Figure 1**

**
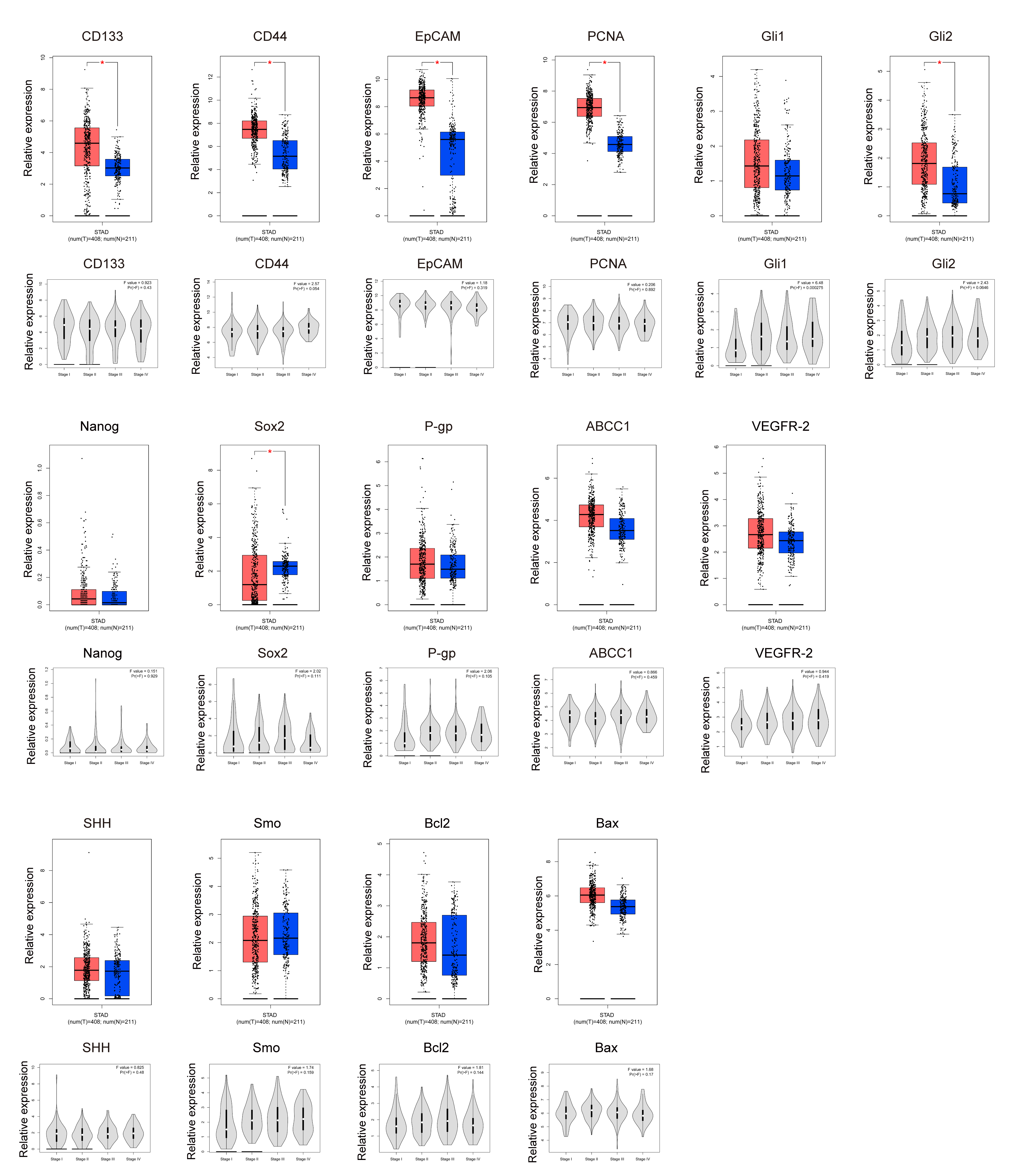
**

**Supplementary Figure 1. Bioinformatics analysis for target genes expression and clinical relevance.** Gene expression of target genes in GC compared with normal tissues using an online TCGA normal and GTEx database (n=211 for normal tissue, n=408 for tumor tissue; one-way analysis of variance). * *p* < 0.05. The expression of target genes in different tumor stages of GC (n=408, one-way analysis of variance) in an online TCGA database.

**Supplementary Figure 2**

**
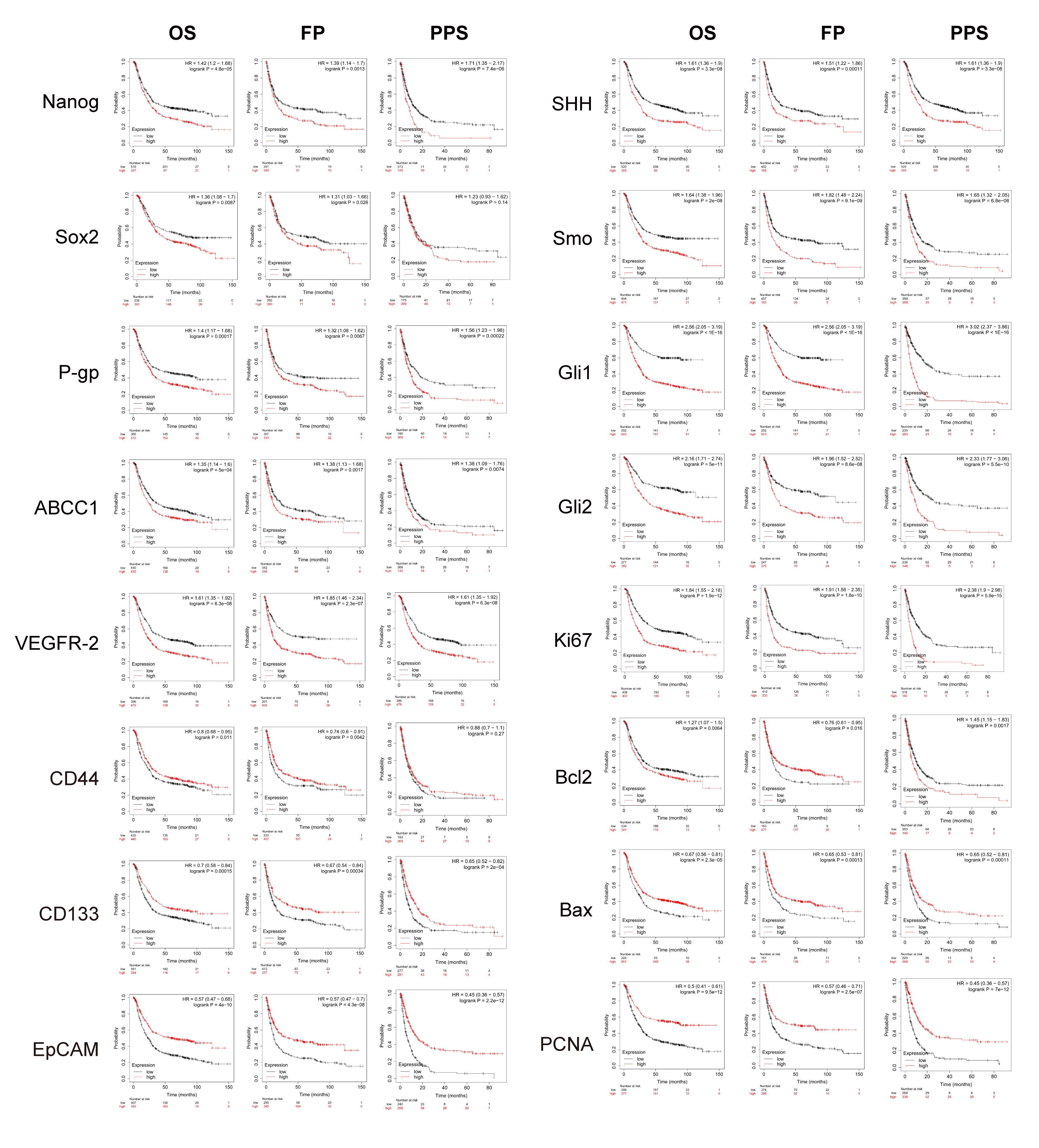
**

**Supplementary Figure 2.** **The prognostic value of mRNA level of related markers in GC patients.** Logrank test was used in analysis of OS (n=875), FP (n=640), PPS (n=498). OS, overall survival; FP, first progression; PPS, post progression survival.

**Supplementary Figure 3**


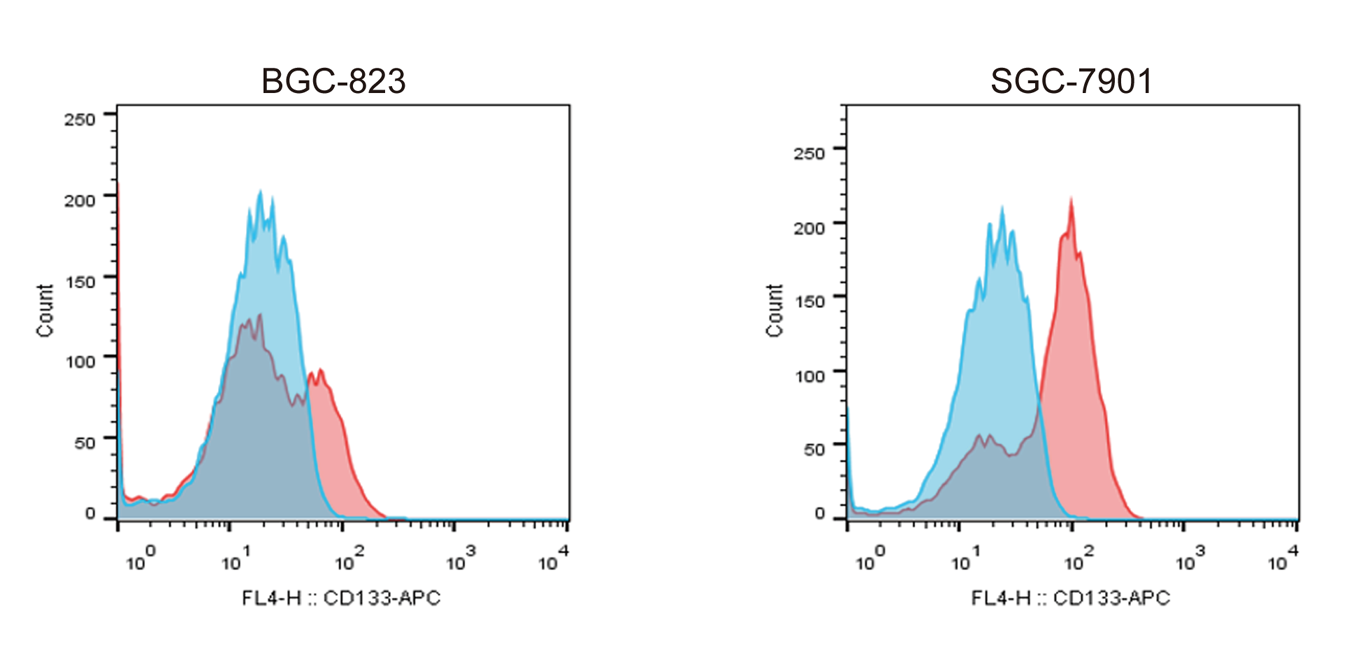


**Supplementary Figure 3.** Detection of CD133-positive cells in both cells.

**Supplementary Figure 4**


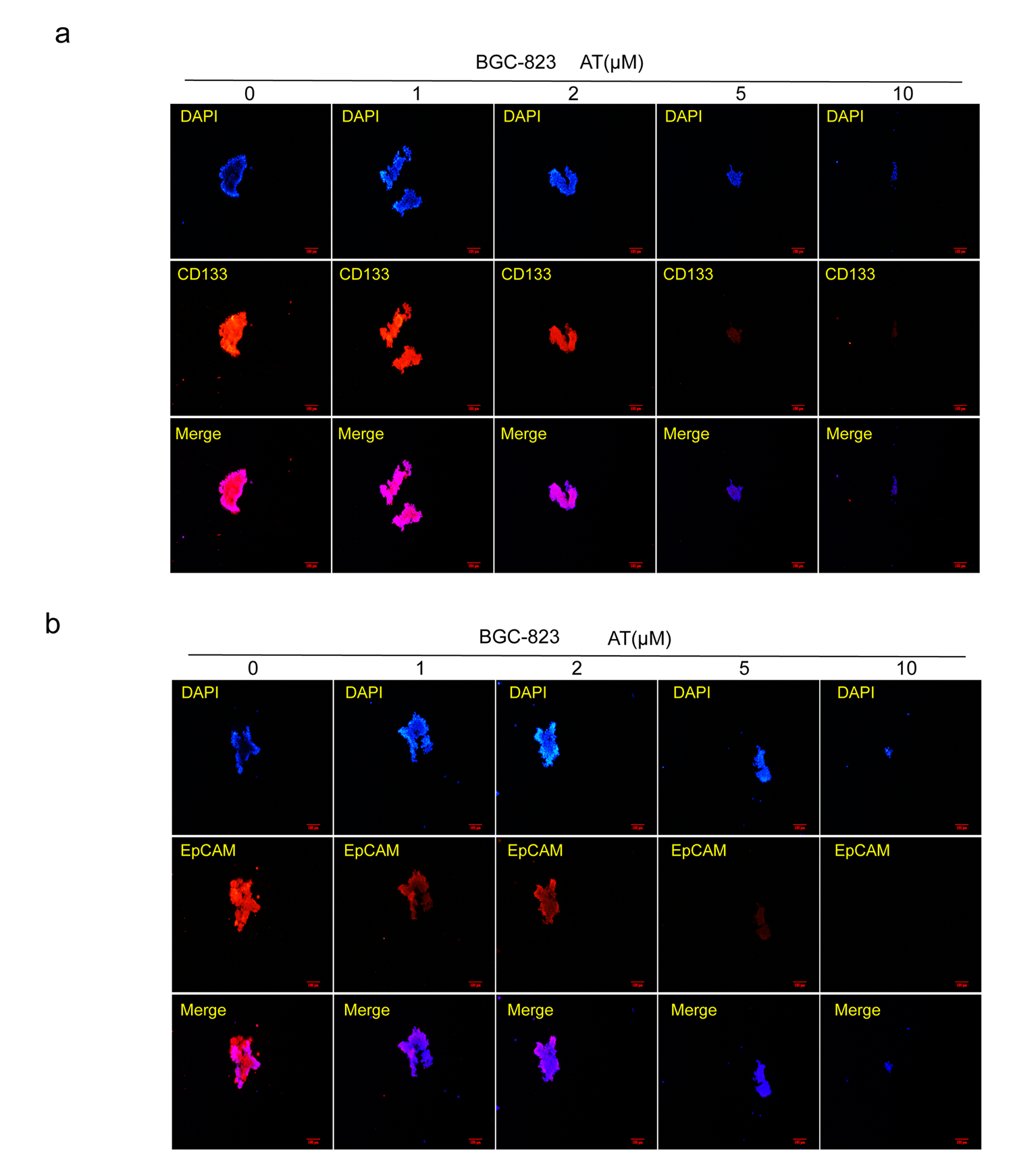


**Supplementary Figure 4. Apatinib inhibits the stemness of GCSCs.** Immunofluorescence staining images of BGC-823 spheroids were obtained to determine the expression of CD133 and EpCAM after apatinib treatment (0,1,2,5, and 10 µM). Bar 100 µm.

**Supplementary Figure 5**

**
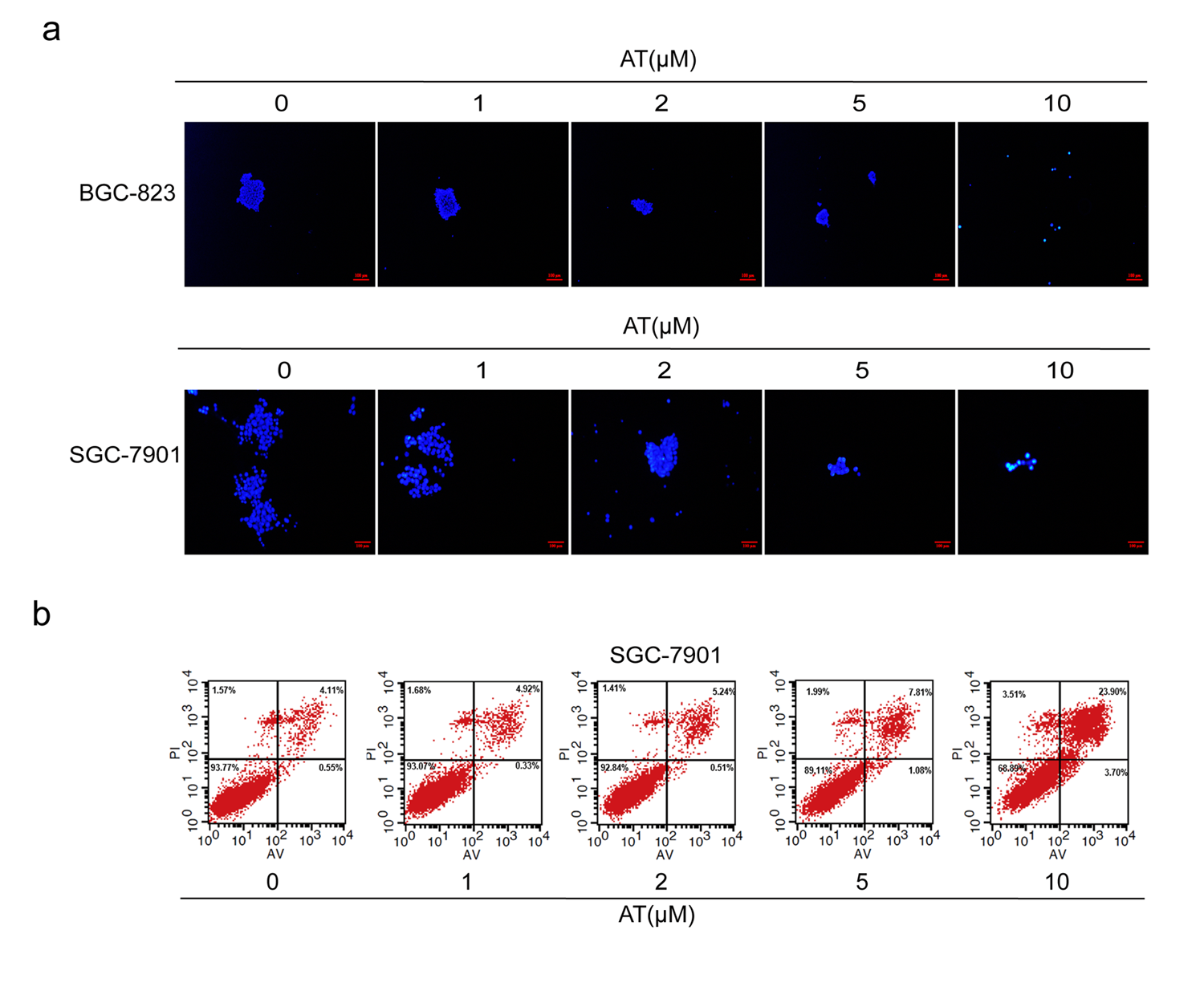
**

**Supplementary Figure 5. Apatinib treatment induces GCSCs apoptosis.** **a** Hoechst 33258 staining of the tumorspheres. Bar 100 μm. **b** The percentage of apoptotic cells was analyzed by flow cytometry.

**Supplementary Figure 6**

**
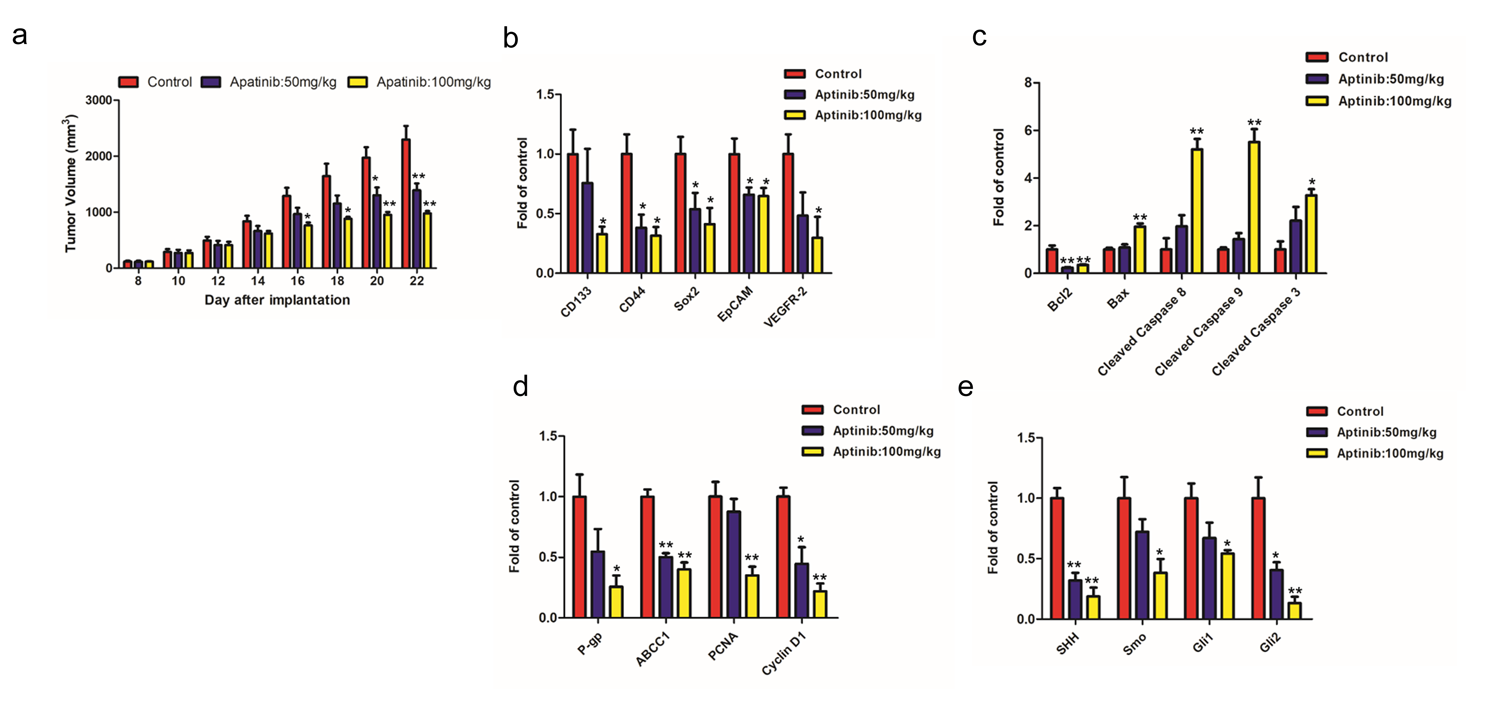
**

**Supplementary Figure 6. Apatinib inhibits GCSCs traits in vivo.** **a** Changes in tumor volume. Data are presented as mean ± SD Changes in body weight. Data are presented as the mean ± SD. **b-e** Densitometric values were quantified by Image J software and normalized to control. The values of control were set to 1.0. The data were presented as means ± SD of three independent experiments. * *p* < 0.05, ** *p* < 0.01 compared with control group.
